# Segmental arterial stiffness in relation to B-type natriuretic peptide with preserved systolic heart function

**DOI:** 10.1371/journal.pone.0183747

**Published:** 2017-09-18

**Authors:** Chih-Hsuan Yen, Chung-Lieh Hung, Ping-Ying Lee, Jui-Peng Tsai, Yau-Huei Lai, Cheng-Huang Su, Hung-I Yeh, Charles Jia-Yin Hou, Kuo-Liong Chien

**Affiliations:** 1 Division of Cardiology, Department of Internal Medicine, Mackay Memorial Hospital, Taipei, Taiwan; 2 Mackay Medicine, Nursing and Management College, Taipei, Taiwan; 3 Institute of Preventive Medicine, School of Public Health, National Taiwan University, Taipei, Taiwan; 4 Department of Medicine, MacKay Medical College, New Taipei City, Taiwan; 5 Department of Internal Medicine (Cardiology), National Taiwan University Medical College and National Taiwan University Hospital, Taipei, Taiwan; Kurume University School of Medicine, JAPAN

## Abstract

**Background:**

Central arterial stiffness has been shown to play a key role in cardiovascular disease. However, evidence regarding such arterial stiffness from various arterial segments in relation to B-type natriuretic peptide (BNP) remains elusive.

**Methods:**

A total of 1255 participants (47.8% men; mean age: 62.6 ± 12.3 [SD] years) with preserved left ventricular function (ejection fraction ≥50%) and ≥1 risk factors were consecutively studied. Arterial pulse wave velocity (PWV) by automatic device (VP-2000; Omron Healthcare) for heart-femoral (hf-PWV), brachial-ankle (ba-PWV), and heart-carotid (hc-PWV) segments were obtained and related to BNP concentrations (Abbott Diagnostics, Abbott Park, IL, USA).

**Results:**

Subjects in the highest hf-PWV quartile were older and had worse renal function and higher blood pressure (all P < 0.05). Elevated PWV (m/s) was correlated with elevated BNP (pg/ml) (beta coefficient = 19.3, 12.4, 5.9 for hf-PWV, ba-PWV, hc-PWV respectively, all p < 0.05). After accounting for clinical co-variates and left ventricle mass index (LVMI), both hf-PWV and ba-PWV were correlated with higher BNP (beta coefficient = 8.3, 6.4 respectively, P < 0.01 for each). Adding both hf-PWV and ba-PWV to LVMI significantly expanded ROC in predicting abnormal BNP>100 pg/ml (both P < 0.01), but only hf-PWV presented significant integrated discrimination improvement to predict risk for BNP concentrations (0.7%, P = 0.029).

**Conclusion:**

A significant segmental PWV associated with biomarker BNP concentrations suggests that arterial stiffness is associated with myocardial damage.

## Introduction

Heart failure continues to be a major and growing public health issue and remains the leading cause of hospitalization; it is associated with an approximately 45% post-discharge mortality and readmission rate within 3 months.[1,2] Despite treatment using pharmacologic and mechanical (device) interventions, the clinical outcome of patients with preserved ejection fraction heart failure (HFpEF) remains suboptimal[3], with a hospitalization rate and poor prognosis[4–6] as malignant as systolic dysfunction heart failure (HFrEF, median survival: 2.1 years). [7] B-type natriuretic peptide (BNP) is a reliable biomarker for diagnosing heart failure and has demonstrated predictive value for hospitalization and mortality both in HFpEF and HFrEF. [8,9]

Pulse pressure (PP) has emerged as a predictor for heart failure in the elderly population. [10,11] Greater afterload from increased arterial stiffness has also been shown to be the key pathophysiology in subjects with known hypertension in the elderly population for diminished arterial compliance, which may cause cardiac dysfunction and contribute to elevated BNP level[12–14]. Among several indices available for clinical use, both aortic pulse wave velocity (PWV) and central carotid-femoral pulse wave velocity (cf-PWV) have been considered for identifying arterial stiffness in an asymptomatic or in relatively high risk population. [15,16] Automatic assessment of brachial-ankle pulse wave velocity (ba-PWV) is an alternative method that takes less time, but its clinical use for prediction of coronary artery disease remains less proven. [17]

Cardiac dysfunction can be caused by several factors including arterial stiffness, systemic inflammation, accumulation of adiposity, and vascular-ventricular overload, and uncoupling. [18–20] Tartiere et al. reported that the central aortic arterial stiffness marker cf-PWV is an independent prognostic factor for HFpEF, [21] and Yambe et al. further demonstrated that higher peripheral muscular arterial stiffness (ba-PWV) is linked to elevated BNP concentration in patients with hypertension.[15] In the current study, we investigated an intermediate- to high-risk population with preserved EF function to determine the relations among PWV of different arterial segments (central aortic and peripheral muscular) and circulating BNP levels, a clinical surrogate of potential myocardial damage. This study also aimed to examine predictive beyond traditional echocardiography-defined left ventricle (LV) geometric parameters, such as left ventricle mass index (LVMI).

## Methods

### Study design and study population

All participants in the tertiary center study were recruited from cardiovascular outpatient clinics at Mackay Memorial Hospital from August 2009 to July 2011. A cross-sectional study was conducted in patients with preserved left ventricular systolic function (LVEF ≥50%) and at least 1 risk factor (e.g., hypertension or diabetes) from the cardiovascular outpatient department. This study was approved by local ethical institutional committee (Mackay Memorial Hospital) for retrospective data analysis without informed consent of study participants. Data security was guaranteed, and all authors had no access to patient identifying information before and after data analysis. Study participants involved in this study were not under clinical service of current study physicians or researchers. In brief, 1500 consecutive patients were enrolled who presented at outpatient clinics with intermediate risk and preserved heart systolic function. A total of 11 patients did not have blood test data, 66 patients did not perform the complete pulse wave velocity exam, 12 patients did not receive clear echocardiography images, and 154 patients did not consent to the BNP blood test. One patient with extremely high BNP concentration (5000 pg/ml) and another with extremely low pulse wave velocity (hf-PWV = 1.19 m/s) were excluded. A total of 1255 patients with complete arterial stiffness exams and BNP data were enrolled in the study. Of the 1255 participants with complete arterial pulse wave velocity exams, 47.8% patients were male with a mean age of 62.6 ± 12.3 (±SD) years. Demographic data and medical history were collected by three independent cardiologists during face-to-face interviews. Anthropometric parameters including height, body weight, and waist circumference were measured by an experienced study nurse. The risk factors were age (male>45 years, female>55 years), high-density lipoprotein cholesterol (male<40 mg/dl, female<50 mg/dl), hypertension, hypercholesterolemia, diabetes mellitus (DM), chronic renal insufficiency (creatinine>1.3 mg/dl), smoking history, and obesity. Hypertension was defined by usage of anti-hypertensive agents or systolic blood pressure (SBP) ≥140 mmHg and/or diastolic blood pressure (DBP) ≥90 mmHg. Hypercholesterolemia was defined by usage of lipid-lowering medicine (statin or ezetamide) or total cholesterol ≥200 mg/dl or low-density lipoprotein cholesterol ≥130 mg/dl. Diabetes was defined by known diabetes history, fasting blood glucose >126 mg/dl or any current usage of DM medication. Smoking history was defined as being an ex-smoker or with current tobacco usage. Patients were considered obese with a body mass index (BMI) > 30 kg/m^2^. Coronary artery disease was defined by known myocardial infarction history or significant coronary stenosis requiring percutaneous intervention, including stent deployment. Patients with a known history of cardiovascular surgery or the presence of rheumatic heart disease, atrial fibrillation, previous implantation of a pacemaker, and overt renal insufficiency (creatinine > 3.0 mg/dl) or with dialysis history were all excluded from the study.

### Echocardiography

Transthoracic echocardiography in two dimensions and Doppler mode examinations were performed on 1371 participants using a commercially available ultrasound system (GE Vivid 7, Vingmed) equipped with a 2–4 MHz transducer. All reported echocardiography measurements are the average value of three successive heart cycles. We assessed parameters of left atrial (LA) diameter, interventricular septal thickness, posterior ventricular wall thickness, left ventricular diameter of both end-diastolic and end-systolic phase, and subsequent LV mass (g) determined from linear M-mode method according to the recommended method established by the American Society of Echocardiography. [22] LV mass index (g/m^2^) was defined as the ratio of LV mass to body surface area (BSA). Gender-specific LV hypertrophy (LVH) was defined by LVMI >95gm/m^2^ for women and > 115gm/m^2^ for men. [22]

### Pulse wave velocity

Arterial stiffness was measured using pulse wave velocity. After resting for 5 min in a supine position, bilateral ba-PWV, systolic blood pressure, and diastolic blood pressure from four limbs were measured simultaneously with an automated machine (VP-2000; Collin Corp., Japan). Carotid artery and femoral artery pulse waveforms were measured via manual recording by a single experienced technician. The central aortic hf-PWV and carotid hc-PWV were measured with a time delay between the rapid upstroke of the heart-femoral artery and the heart-carotid artery and then calculated by an automatic device and manual measurement (VP-2000; Collin Corp., Komaki Japan). PWV was calculated as the distance between the two arterial sites divided by the time delay between the two arterial point sites and presented as centimeter per seconds. [20]

### Laboratory measurements

Overnight fasting blood serum and plasma samples were collected to determine glucose and lipid profiles (total cholesterol, triglyceride, low-density lipoprotein cholesterol, and high-density lipoprotein cholesterol). Some biochemical measurements, including renal function, were also obtained. Serum samples were collected using standard sampling tubes or tubes containing separating gel. After ensuring individualized patient samples, calibrators and controls were set at ambient temperature (20°C–25°C), and the measurement was taken within 2 h. Hs-CRP level was determined by a highly sensitive, latex particle-enhanced immunoassay using Elecsys 2010 (Hitachi Corp. Hitachinaka Ibaraki, Japan). Serum BNP concentrations were measured using a fluorescence immunoassay microtiter plate with a coefficient of variation of 10.4% (Alere Biosite Triage, San Diego, CA, USA). Renal function was determined by estimating the glomerular filtration rate (eGFR), which was calculated using the Modification of Diet in Renal Disease formula in a Chinese population: eGFR (ml/min/1.73 m^2^) = 175 × P_Cr_ (plasma creatinine, mg/dl) ^−1.234^ × age ^−0.179^ × (0.79 if woman). [23]

### Statistical analysis

Participants were categorized on the basis of quartile of hf-PWV level, and continuous variables were presented as mean ± SD. Categorical data were presented as proportions or percentage rate. A nonparametric method (Mann–Whitney U test) was used to test for continuous variables among the quartile and chi square or Fisher’s exact test method were used to compare categorical variables. The relationship between BNP and PWV was examined by age- and gender-adjusted Spearman’s partial correlation coefficients. Multiple logistic regression analyses were conducted to measure different PWVs associated with BNP concentrations independent of other clinical parameters. A receiver operating characteristic (ROC) curve was used to evaluate the area under the curves to identify abnormally high BNP (BNP>100 pg/ml) with c-statistics used for comparison between different models. For reclassification analysis, cross-classification was measured separately in participants with higher BNP concentrations (>100 pg/ml) and its lower parts (≤100 pg/ml). Integrated discrimination improvement is equal to the difference in discrimination slopes between the old model and a new PWV predicting model. The reproducibility of PWV measurement was presented using the Bland–Altman plot. All statistical tests were tow-tailed, and P < 0.05 was considered statistically significant. Analyses were performed with Stata version 11 (Stata Corp., College Station, TX, USA) and SAS 9.2 version (SAS Institute, Cary, NC, USA).

## Result

**Fig 1:**
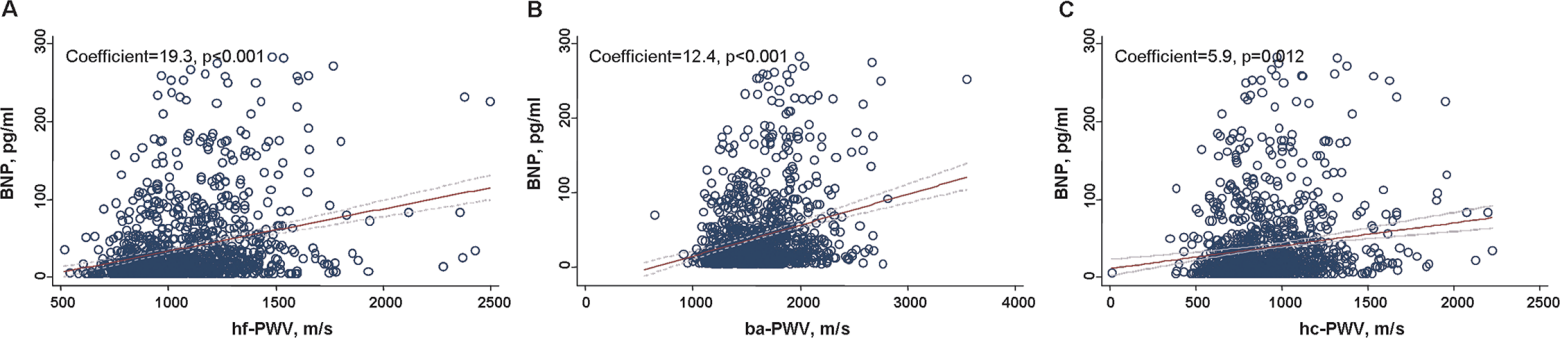
Linear relationship as regression and scatter plots among circulating BNP level, ba-PWV, hf-PWV, and hc-PWV.

All 1409 (mean: 62.6 ± 12.3, range of age: 20–88 years) participants were stratified by hf-PWV level. Participants in the highest quartile were older, had worse renal function, and had higher blood pressure; their serum BNP levels were comparable with those patients in the lowest quartile (P < 0.05 for each; Table 1). The correlations among the hf-PWV, ba-PWV, and hc-PWV levels were 0.44 and 0.53 (P < 0.001 for each). The Spearman’s correlation rho between BNP and hf-PWV, ba-PWV, and hc-PWV were 0.28, 0.23, and 0.16, respectively (P < 0.001 for each). Elevated PWV was correlated with higher BNP levels (coefficient = 19.3, 12.4, and 5.9 for hf-PWV, ba-PWV, and hc-PWV respectively; P < 0.05 for each), and hf-PWV was strongly associated with elevated serum BNP (r = 0.28, P < 0.01; Fig 1). Pearson correlation showed that greater left ventricular EF was inversely associated with BNP level (R = −0.08, p = 0.006), and large LA diameter and LVMI were positively associated with higher BNP (r = 0.16 & 0.17, both p < 0.001). Subjects with higher blood pressure (defined as SBP ≥140 mmHg and/or DBP ≥90 mmHg) or abnormally high BNP (>100 pg/ml) were more likely to present with LVH (37% vs. 25%, *X*^2^ = 0.002).

**Table 1:** Descriptive quartile of heart-femoral pulse wave velocity (hf-PWV) (m/s).

Data range of hf-PWV, range	Q1 (5.1–9.0)	Q2 (9.0–10.4)	Q3 (10.4–12.2)	Q4 (12.2–24.9)	
Total N = 1409	N = 352	N = 358	N = 348	N = 351	P value
Age, years, mean ± SD	53.8 ± 10.4	60.1 ± 9.9	64.5 ± 10.3	70.8 ± 11.0	<0.001
Gender, female, n (%)	152(43%)	156(44%)	187(54%)	174(50%)	0.012
Systolic Blood pressure, mmHg	123 ± 16.7	130.1 ± 17.2	138.1 ± 18.6	146.2 ± 20.4	<0.001
Diastolic Blood pressure, mmHg	73.5 ± 10.9	76.8 ± 11.4	78.9 ± 11.9	80.5 ± 12.5	<0.001
Body Mass Index, kgw/m^2	26.2 ± 4.1	25.7 ± 3.9	25.7 ± 3.7	25.4 ± 3.8	0.018
Obesity, n (%)	60(17%)	46(13%)	42(12%)	39(11%)	0.09
Left atrial (LA) diameter, mm	31.6 ± 5.2	32.5 ± 4.9	33.6 ± 4.8	35.1 ± 6.0	<0.001
Left ventricular EF, %	64.4 ± 6.8	64.4 ± 6.5	62.8 ± 6.5	63.5 ± 6.6	0.009
Left ventricular mass, g	153 ± 40	162 ± 44	166 ± 54	174 ± 49	<0.001
Left ventricular mass index, g/BSA	90.1 ± 20.7	96.5 ± 24.4	98.0 ± 30.7	104.6 ± 25.8	<0.001
Smoker, n (%)	83(24%)	80(22%)	98(28%)	74(21%)	0.13
Hypertension, %	225(64%)	275(77%)	285(82%)	271(77%)	<0.001
Diabetes, %	83(24%)	95(27%)	117(34%)	167(48%)	<0.001
Coronary artery disease, %	85(24%)	77(22%)	81(23%)	92(26%)	0.53
Heart failure history, %	24(9%)	33(12%)	30(12%)	47(19%)	0.006
Stroke, %	3(0.8%)	4(1%)	9(2%)	21(6%)	<0.001
Chronic renal insufficiency, n (%)	12(3%)	23(6%)	33(9%)	78(22%)	<0.001
Fasting sugar, mg/dl	108.6 ± 28.3	115.4 ± 40.7	117.1 ± 32.9	129.6 ± 49.4	<0.001
Cholesterol, mg/dl	197.1 ± 41.1	201.3 ± 43.2	194.2 ± 43.4	183.9 ± 43.3	<0.001
TG, mg/dl	140.5 ± 110.5	145.8 ± 122.9	141.1 ± 101.5	144.3 ± 97.8	0.19
LDL-C, mg/dl	118.1 ± 35.6	121.7 ± 33.7	116.1 ± 36.8	110.9 ± 34.4	<0.001
HDL-C, mg/dl	47.2 ± 13.8	47.7 ± 14.8	46.4 ± 13.9	45.7 ± 17.2	0.01
Creatinine, mg/dl	0.8 ± 0.3	0.8 ± 0.3	0.9 ± 0.3	1.1 ± 0.6	<0.001
eGFR, ml/min/1.73 m^2	99.9 ± 28.1	89.5 ± 26.1	81.4 ± 24.8	70.7 ± 29.7	<0.001
BNP, pg/ml	47.9 ± 161.8	51.6 ± 132.2	59.6 ± 110.3	154.9 ± 339.5	<0.001
hs-CRP, mg/dl	0.24 ± 0.4	0.28 ± 0.6	0.38 ± 0.8	0.51 ± 1.1	<0.001
Drugs					
Aspirin, %	88(25%)	100(28%)	94(27%)	91(26%)	0.81
Clopidogrel or Panaldin, %	11(3%)	22(6%)	23(7%)	49(14%)	<0.001
ACE inhibitor, %	19(5%)	34(10%)	32(9%)	35(10%)	0.11
ARB, %	129(37%)	152(43%)	167(48%)	187(53%)	<0.001
Calcium-channel blocker, %	156(44%)	185(53%)	185(53%)	212(60%)	<0.001
Statin, %	106(30%)	133(37%)	126(36%)	121(35%)	0.19
Diuretics, %	53(15%)	76(21%)	98(28%)	121(35%)	<0.001
Beta-blockade, %	188(53%)	203(57%)	197(57%)	193(55%)	0.72

Both hf-PWV and ba-PWV were associated with elevated serum BNP levels (beta coefficient = 8.3 and 6.4, respectively, both P < 0.01; Fig 1) after using a multi-variable regression model that adjusted for age, gender, BMI, systolic blood pressure, PP, renal function, LVMI, left ventricular ejection fraction (EF), and coronary artery disease (S1 Table.). However, both hf-PWV and ba-PWV levels were not significantly associated with predicting categorical BNP (hf-PWV and ba-PWV cut-point = 12.2 and 18.1 m/s, respectively; BNP cut-point = 100 pg/ml; AOR = 1.43 and 1.34, respectively; and P = 0.1 and P = 0.16, respectively) (Tables 2 and 3).

**Table 2:** The mean ± SD, numbers of the study participants, and ORs by hf-PWV or ba-PWV quartile for the association with BNP (cut-off level 100 pg/ml) in the study participants.

2–1					
			hf-PWV quartile		
	1	2	3	4	P for trend
Mean ± SD hf-PWV, m/s	8.1 ± 0.7	9.7 ± 0.3	11.3 ± 0.5	14.4 ± 2.3	
Participants, n	352	358	348	351	
OR, model 1*^a^	1	1.01 (0.54–1.84)	0.93 (0.50–1.71)	1.67 (0.92–3.03)	0.09
OR, model 2*^b^	1	1.08 (0.59–2.31)	0.83 (0.43–1.73)	1.36 (0.68–2.85)	0.37
OR, model 3*^c^	1	1.17 (0.41–1.51)	0.86 (0.55–1.85)	1.39 (0.67–2.32)	0.35
2–2					
			ba-PWV quartile		
	1	2	3	4	P for trend
Mean ± SD ba-PWV, m/s	12.5 ± 1.0	14.8 ± 0.5	16.8 ± 0.7	20.7 ± 3.0	
Participants, n	371	366	368	368	
OR, model 1*^a^	1	0.66 (0.37–1.19)	0.83 (0.49–1.42)	1.13 (0.67–1.91)	0.64
OR, model 2*^b^	1	0.76 (0.41–1.42)	0.96 (0.53–1.71)	1.22 (0.67–2.21)	0.51
OR, model 3*^c^	1	0.79 (0.41–1.51)	1.01 (0.55–1.85)	1.24(0.67–2.32)	0.48

* Data for OR (95% CI) are expressed relative to hf-PWV quartile 1

^a^ Model 1: Adjusted for age, gender

^b^ Model 2: Model 1 plus gender, body mass index (BMI), systolic blood pressure, pulse pressure, renal function (eGFR), coronary artery disease history(yes/no)

^c^ Model 3: Model 2 plus left ventricle mass index, left ventricle ejection fraction

**Table 3:** The chosen cut-offs, numbers of the study participants, and ORs by higher and lower hf-PWV or ba-PWV for the association with BNP (cut-off level 100 pg/ml) in the study participants.

3–1			
	Lower	Higher	P for trend
Cut-off hf-PWV, m/s	< = 12.2	>12.2	
Participants, n	1058	351	
OR, model 1*^a^	1	1.73 (1.18–2.52)	0.004
OR, model 2*^b^	1	1.47 (0.96–2.25)	0.07
OR, model 3*^c^	1	1.43 (0.93–2.22)	0.1
3–2			
	Lower	Higher	P for trend
Cut-off ba-PWV, m/s	< = 18.1	>18.1	
Participants, n	1106	365	
OR, model 1*^a^	1	1.41 (0.98–2.03)	0.06
OR, model 2*^b^	1	1.37 (0.91–2.05)	0.12
OR, model 3*^c^	1	1.34 (0.88–2.04)	0.16

* Data for OR (95% CI) are expressed relative to lower hf-PWV quartile

^a^ Model 1: Adjusted for age, gender

^b^ Model 2: Model 1 plus gender, body mass index (BMI), systolic blood pressure, pulse pressure, renal function (eGFR), coronary artery disease history(yes/no)

^c^ Model 3: Model 2 plus left ventricle mass index, left ventricle ejection fraction

**Fig 2:**
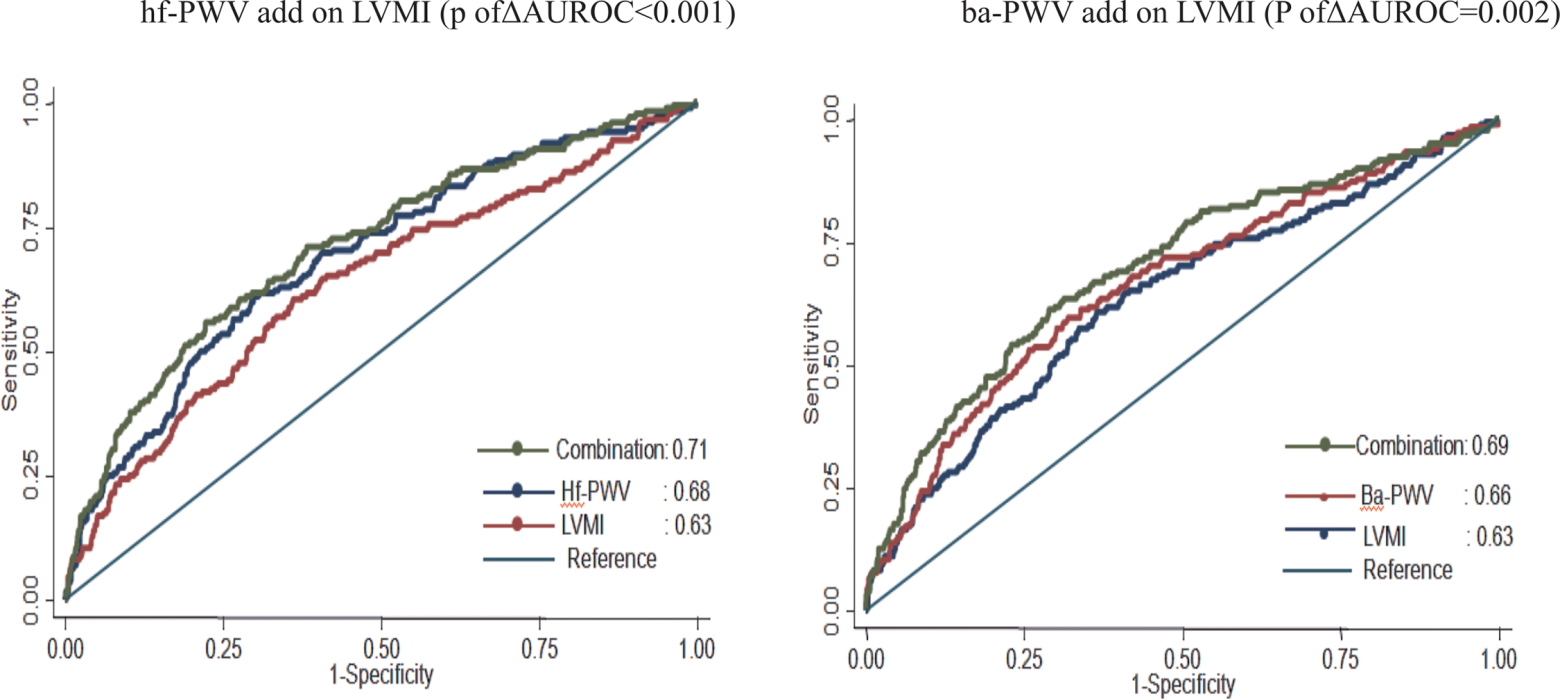
ROC curve for hf-PWV and ba-PWV superimposed on LVMI in predicting abnormally high BNP (>100 pg/ml).

Adding both hf-PWV and ba-PWV to LVMI significantly added the C-statistics area under the ROC from 0.63 to 0.71 and from 0.63 to 0.69, respectively (P < 0.01 for each), in predicting high-risk heart failure (BNP>100 pg/ml; Fig 2). Furthermore, the addition of hf-PWV, ba-PWV, and hc-PWV did not result in an incremental predictive value for high BNP (>100 pg/ml) when using the basic model, which adjusted for age, gender, BMI, systolic blood pressure, PP, and LVMI (P of ΔAUROC = 0.66; Fig 2). Meanwhile, the addition of hf-PWV to the basic model resulted in an upward reclassification of 6.3%, as well as an 8.4% downward reclassification, yielding an overall net reclassification of −2.1% of participants who had abnormally high BNP (>100 pg/ml) concentrations. For participants who had a lower BNP (≤100 pg/ml), addition of hf-PWV resulted in an upward reclassification of 3.7% and a downward reclassification of 6.2%, which yielded an overall net reclassification of 2.5%. In the full sample, when hf-PWV was added to the basic model, we observed a net reclassification index of 0.4% (95% CI, −5.2% to 6.1%; P = 0.87) and an integrated discrimination improvement of 0.7% (95% CI, 0.02% to 1.4%; P = 0.029; Table 4)

**Table 4:** Predicted risk for BNP concentrations before and after reclassification with hf-PWV in participants who with higher (>100 pg/ml)(A) and lower (< = 100 pg/ml)(B).

			Model with hf-PWV		
A	0–10%	10–15%	15–20%	>20%	Total
Model without hf-PWV					
0–10%	42*	3	0	0	45
10–15%	3	5*	3	1	12
15–20%	0	3	12*	5	20
>20%	9	0	1	103*	113
Total	54	11	16	109	190
B	0–10%	10–15%	15–20%	>20%	Total
Model without hf-PWV					
0–10%	773*	11	0	1	785
10–15%	30	127*	17	1	175
15–20%	10	14	54*	18	96
>20%	18	0	9	204*	231
Total	831	152	80	204	1287

* These values along the diagonal were similarly classified by both models. Values on each row to the right of the value with an asterisk were upwardly classified, and those to the left were downwardly classified by the model that included hf-PWV, Net reclassification improvement 0.0045 (CI. −0.052, 0.061, P = 0.87); Integrated discrimination improvement.0072 (CI. 0.0002, 0.014, p = 0.029)

## Discussion

### Pulse wave velocity and BNP concentrations

Although both hf-PWV and ba-PWV were associated with BNP concentrations after adjusting for systolic blood pressure and other clinical factors. Only hf-PWV was associated with a significant improvement in the prediction of BNP in additional basic clinical parameters in individuals with baseline preserved global EF. The clinical diagnosis of HFpEF as determined by BNP and heart echocardiography parameters have been well established and supplied as useful diagnostic and prognostic tools. [8,24] In our current work, pulse wave velocity after central aortic segments substantially improved the predictive power to identify abnormally high BNP concentrations, which may help identify a specific target group of patients at higher risk of HFpEF development from increased vascular stiffness as the main underlying pathophysiology. Therefore, recognition of such a population may potentially benefit from early therapeutic interventions.

### Segmental PWV: Central aortic PWV, muscular arterial PWV, and carotid arterial PWV

Assessment of arterial stiffness has revealed a strong relationship between stiffness of the central carotid artery and the abdominal aorta and increased cardiovascular event rates in many high-risk[25–27] and community-based populations. [28–30] Several methods have been evaluated in clinical trials, and PWV was considered the gold standard among other non-invasive methods. [11–31] Peripheral ba-PWV was assessed at two sites between the brachial artery and the ankle anterior tibial artery; [32] meanwhile, the central cf-PWV was evaluated at two sites between the carotid artery and the femoral artery. [33] In recent literature, ba-PWV was shown to correlate better with cardiac and vascular structure and function compared with cf-PWV, especially in Asians. [17,34] Further, the central arterial stiffness measurement is known to be skill-dependent, whereas PWV measurement is automatic and represents the PWV for the entire central and peripheral arterial system with a paucity of outcome data. [35] Compared with Kang’s report on ba-PWV measures (15.1 m/s) in middle and aged populations with diastolic hear failure, our cohort presented close values (18.1 m/s) of ba-PWV. [35] Thus, we speculated that PWV may be an alternative convenient and non-invasive measure, which may provide additional information for cardiac dysfunction in phenotypic HFpEF reflected by elevated BNP level, especially in Asian populations. [36] In our current study, we demonstrated that both the ba-PWV and central hf-PWV were useful for BNP concentration correlation, although the central aortic arterial stiffness (hf-PWV) was more closely correlated with BNP level.

### Biological mechanism

In this study, measures of arterial stiffness (hf-PWV and ba-PWV) may provide additional information beyond degree of ventricular remodeling. Both high degree of LV remodeling in terms of large LVMI and large LA diameter were linked to elevated BNP, thereby reflecting the LV remodeling process and diastolic dysfunction. Moreover, aortic arterial stiffness (hf-PWV) had an additional risk reclassification effect in identifying the cardiac damage risk model, and it was defined by an abnormally high BNP level (>100 pg/ml). In general, central aortic elastic compliance supplied a cushioning effect during heart diastole and maintained the pulse processing from central to downstream sites. High baby birth weight is positively associated with greater central aortic stiffness, implying that impaired glucose metabolism may play an important role. [37]

The carotid artery remodeling proximal to the heart from adaptative remodeling of increased wall tension in greater arteries is tightly linked to load status, such as blood pressure or PP. [38] The diameter of the carotid artery was related to BNP concentrations in patients with heart failure within a preserved EF population. [39] However, given that the distance between the heart and carotid artery was too short to result in the stiffness effect, passing through the aorta to the femoral artery was more effective in predicting BNP levels.

### Pulse wave velocity, BNP, preserved EF heart failure, and treatment

Increasing rates of preserved EF heart failure within the aging society is an important problem that will become an economic burden in the near future^1^. The coupling of ventricular arterial stiffness with mechanical interaction of systemic and coronary flow balance, regulation of endothelial function, and smooth muscle tone implies the importance of vascular pressure load to heart function. [40,41] In early studies in Japan and China, arterial stiffness was considered a risk factor for both atherosclerosis and diastolic heart failure but not the adjustment of renal function. [34,42] A recent Russian study disclosed that considerable arterial stiffness was increased in patients with preserved EF heart failure after ST elevation and acute myocardial infarction.[43] In our study, we discovered that both hf-PWV and ba-PWV were associated with BNP concentrations after adjusting for renal function. However, high hf-PWV was not correlated with a high prevalence rate of coronary heart disease in a preserved EF population, thereby implying that arterial stiffness had another mechanism for inducing heart damage beyond coronary injury.

Arterial stiffness was the consequence of progressive arterial dilation and degeneration of the arterial wall, and it occurs alongside aging and increased systolic pressure. Furthermore, peripheral brachial systolic pressure impaired systolic pressure in the aorta and left ventricle, thereby inhibiting the vessel dilation effect of anti-hypertensive drugs. [44] In addition to the effects of advanced age and the hypertensive status on arterial wall elasticity, some evidence of atherosclerosis implied that this hardening affected the distributed compliance model of arterial circulation, which implied statin utility in elevated PP and arterial stiffness. [45]

### Strength and limitations

In this study, we determined the central aortic and peripheral muscular arterial stiffness indicators (hf-PWV and ba-PWV). Our current study simultaneously measured central aortic (hf-PWV), heart carotid (hc-PWV), and peripheral muscular (ba-PWV) arterial stiffness. Results demonstrated a relationship between hf-PWV and BNP levels even after adjusting for clinical and epidemiologic factors. Although these results create a large database for the use of arterial stiffness in predicting BNP concentration, the study’s generalizability was limited to outpatient clinic patients, so it may not be applicable to community-based populations. Furthermore, we used indirect methods to evaluate arterial stiffness, and data were restricted to a single center. Finally, this work was a cross-sectional study, and further research to confirm this conclusion in longitudinal outcome-driven follow up may be warranted in the future.

## Conclusion

Our study showed a significant segmental PWV associated with BNP concentrations, thereby implying that arterial stiffness was related to cardiac damage. Adding on geometric LVMI, hf-PWV, and ba-PWV significantly increased our ability to predict heart damage. Only central aortic hf-PWV significantly improved our ability to predict BNP concentrations, and this parameter was correlated with aortic arterial stiffness and worsening heart function. Further study and evaluations are warranted.

## Supporting information

S1 Table Uni-variable and multi-variable linear regression between B-type natriuretic peptide (BNP) level and different PWV values. (DOC)
