# Supplementary material for: Segmental arterial stiffness in relation to B-type natriuretic peptide with preserved systolic heart function
Source: PLoS One. 2017 Sep 18;12(9):e0183747. doi: 10.1371/journal.pone.0183747 (PMC5602521; doi:10.1371/journal.pone.0183747)
Supplement: S1 Table — (DOC) [file pone.0183747.s001.doc]

**Supplemental Materials**

**Table 1. Uni-variable and multi-variable linear regression between B type natriuretic peptide (BNP) level and different PWV values**

| **Predictor** | **Coefficients** | **95% C.I.** | **P-value** |
| --- | --- | --- | --- |
| **hf-PWV, m/s** | 19.3 | 14.9-23.7 | <0.001 |
| **ba-PWV, m/s** | 12.4 | 8.6-16.2 | <0.001 |
| **hc-PWV, m/s** | 5.9 | 1.3-10.6 | 0.012 |

| **Predictor** | **Model 1** |  | **Model 2** |  | **Model 3** |  |
| --- | --- | --- | --- | --- | --- | --- |
|  | **Coefficients** | **P-value** | **Coefficients** | **P-value** | **Coefficients** | **P-value** |
| **hf-PWV, m/s** | 15.1 | <0.001 | 9.9 | 0.003 | 8.3 | **0.003** |
| **ba-PWV, m/s** | 7 | <0.001 | 6.7 | 0.003 | 6.4 | **0.004** |
| **hc-PWV, m/s** | 0.8 | 0.74 | -1.3 | 0.57 | -2.6 | 0.28 |

Model 1: Adjusted for age, gender

Model 2: Model 1 plus systolic blood pressure, pulse pressure, body mass

Index (BMI) , renal function (eGFR), coronary artery disease history(yes/no)

Model 3: Model 2 plus left ventricle mass index, left ventricle ejection fraction
